# Supplementary material for: Content-rich biological network constructed by mining PubMed abstracts
Source: BMC Bioinformatics. 2004 Oct 8;5:147. doi: 10.1186/1471-2105-5-147 (PMC528731; doi:10.1186/1471-2105-5-147)
Supplement: Additional File 2 — The original results of the above study (non-essential files are deleted to keep the file size under the limit set by BMC bioinformatics). [file 1471-2105-5-147-S2.bz2 › chilibotAdditionalFile2/dip10/420ID7890768E10289/html/ACVR2_INHA.html]

 


 **ACVR2** and **INHA** 
  
Found 28 abstracts in PubMed, retrieved 10.  
 

 What does Google say? 
 PDF only 
| .edu only 

---

**Interactive relationship** (e.g. stimulation, inhibition, etc)

**Non-interactive relationship** (e.g. studied together, co-existance, homology, etc.)

- Complementary DNA samples produced by reverse transcription were used in the real time PCR to quantify the expression of inhibin alpha  [ **INHA** ]  subunit, inhibin activin beta A, beta B, beta C subunits, follistatin, ACTRIA, ACTRIB, ACTRIIA  [ **ACVR2** ] , ACTRIIB, beta glycan and GAPDH genes.  Ref: 12651901 Mol Hum Reprod, 2003
- The objective of this study was to determine effects of postnatal age on expression of follistatin, inhibin alpha  [ **INHA** ]  subunit, betaA subunit, betaB subunit, and activin receptors Type IA ActRIA, Type IB ActRIB and Type II ActRII  [ **ACVR2** ]  in the developing ovine uterus.  Ref: 12748120 Biol Reprod< MedlineTA>Biol Reprod, 2003
